# Natural History of Airway Hyperresponsiveness and Its Association With Asthma Traits

**DOI:** 10.1111/all.70006

**Published:** 2025-08-22

**Authors:** Sophie Carra, Hongmei Zhang, Ramesh J. Kurukulaaratchy, Syed Hasan Arshad

**Affiliations:** ^1^ Clinical and Experimental Sciences, Faculty of Medicine University of Southampton Southampton UK; ^2^ Division of Epidemiology, Biostatistics, and Environmental Health Sciences, School of Public Health University of Memphis Memphis Tennessee USA; ^3^ The David Hide Asthma and Allergy Research Centre St Mary's Hospital Newport UK; ^4^ Respiratory Biomedical Research Centre University Hospital Southampton Southampton UK

**Keywords:** airway hyperresponsiveness, asthma, bronchial challenge test, methacholine, wheeze

## Abstract

**Background:**

The natural history of airway hyperresponsiveness (AHR) from childhood to adulthood and its association with asthma status are poorly understood. We aim to define the natural history of AHR in relation to asthma characteristics such as symptoms, atopy and lung function to improve our understanding of the changes in AHR with asthma pathophysiology during adolescence.

**Methods:**

Methacholine bronchial challenge test (BCT) was undertaken in the Isle of Wight whole population birth cohort at 10 years (*n* = 783), 18 years (*n* = 585) and 26 years (*n* = 86). Data on wheeze, lung function, and atopy were collected at each time point. Definite AHR was defined as methacholine concentration provoking a 20% decrease in Forced Expiratory Volume in 1 s (PC_20_) at < 4 mg/mL.

**Results:**

AHR prevalence was 21.6% (169/783) at 10 years and 5% (29/585) at 18 years of age (*p* < 0.01). In 406 participants, where methacholine BCT was performed at both 10 and 18 years, 80.9% of those with AHR at age 10 became negative at 18 years. At a population level, AHR trajectory was in the opposite direction to that of asthma (14.7% at age 10 to 17.6% at age 18; *p* = 0.004), atopy (26.9% at age 10 to 41.5% at age 18; *p* < 0.001) and airway obstruction (FEV_1_/FVC ratio of 0.88 at age 10 to 0.87 at age 18; *p* < 0.001). AHR prevalence remained stable between the ages of 18 and 26 years.

**Conclusion:**

The natural history of AHR is characterised by a marked decrease in prevalence during adolescence, in contrast to asthma and other asthma characteristics. Age should be considered when interpreting AHR as an asthma defining trait.

## Graphical abstract

AHR improves in most children over adolescence when its relationship with asthma changes. AHR is better at ruling out asthma in children, while it is superior at ruling in asthma in young adults. Age should be considered when interpreting AHR, and different cutoffs might be required for children and adults.

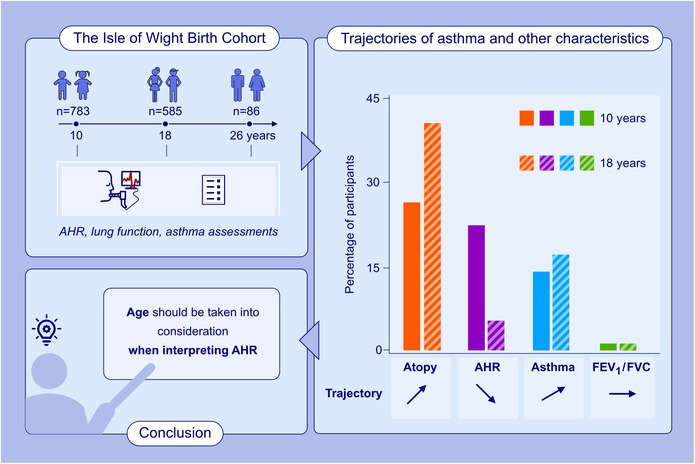

**Abbreviations**
AHR
: airway hyperresponsiveness
FEV_1_ /FVC
: forced expiratory volume in one second to forced vital capacity ratio

## 1 Introduction

Airway hyperresponsiveness (AHR) describes an excessive contraction of bronchial smooth muscle in response to various inhaled stimuli [1]. It is considered a cardinal feature of asthma alongside symptoms, airway inflammation and airflow obstruction with bronchodilator reversibility (BDR) [2]. AHR may be assessed ‘directly’ by stimulation of muscarinic, histamine or other receptors in airway smooth muscle or ‘indirectly’ via stimuli causing release of inflammatory mediators such as airway cooling and drying (e.g., exercise), adenosine monophosphate, or mannitol [3]. The former are regarded as better at ruling out asthma, and the latter are better at ruling it in [4]. Historically, methacholine challenge has been the most used bronchial challenge test (BCT).

Objective confirmation of asthma diagnosis relies on demonstration of reversible airway obstruction. However, given day‐toto‐day and diurnal variability in asthma pathophysiology, a lack of airway obstruction does not exclude asthma. For individuals with suspected asthma who do not show airflow obstruction or BDR, demonstration of AHR may aid in asthma diagnosis as an objective test [5]. Indeed, Louis et al. compared BDR and AHR for asthma diagnosis and demonstrated a superiority for AHR testing [6]. Further, a European Respiratory Society (ERS) Task Force recently suggested that BCT should be performed in secondary care to confirm asthma diagnosis when doubt remains after initial assessment [7].

We, and others, have shown that AHR may be present in those with rhinitis, smokers and even in asymptomatic individuals [8]. Previous studies have estimated the prevalence of AHR to be around 20%–30% in geographically diverse childhood populations [9, 10] and around 10%–20% in adult populations [11]. Thus, AHR might improve in some children as they go through adolescence. However, the natural history of AHR from childhood to later adulthood, along with the longitudinal association of AHR with wheeze and asthma across the life course, is poorly defined. Though traditionally regarded as a diagnostic trait for asthma, it is unclear if, and how, these relationships change from childhood to adulthood. This requires AHR to be studied in an unselected cohort using the same methodology at multiple time points.

A better understanding of the evolution of AHR during adolescence and the resulting changing association with wheeze and asthma could provide valuable insights into its relevance as a characteristic trait in asthma and of methacholine BCT as a tool for asthma assessment and diagnosis at different time points.

In this paper, we studied participants in the Isle of Wight Whole Population Birth Cohort (IOWBC) who had BCT to methacholine to (a) identify the prevalence of AHR at 10 and 18 years, (b) define the natural history of AHR from 10 to 18 years, (c) extend the investigation of AHR natural history into young adulthood (in a subgroup) and (d) study the association of AHR with wheeze/asthma and other recognised asthma characteristics.

## 2 Methods

### 2.1 The Study Cohort and Measurements

In 1989, a whole population birth cohort was established on the Isle of Wight, United Kingdom, to investigate the life course of asthma and allergic disorders. Ethical approval was obtained from the local research ethics committee. Out of the 1536 children born and recruited between January 1, 1989, and February 28, 1990, 1456 participants consented for longitudinal follow‐up and were assessed at ages 1 (*n* = 1167), 2 (*n* = 1174), 4 (*n* = 1218), 10 (*n* = 1373), 18 (*n* = 1313) and 26 (*n* = 1034) years.

Both study‐specific and International Study of Asthma and Allergies in Childhood questionnaires [12] were completed for a detailed assessment of asthma symptoms and treatment. Skin prick tests (SPTs) were performed on most children attending the research centre at ages 4 (*n* = 982), 10 (*n* = 1036) and 18 (*n* = 853) years to determine their allergic sensitization status. A standard battery of common allergens (ALK‐Albello, Horsholm, Denmark) was tested. Allergens tested were house dust mite (Dermatophagoides pteronyssinus), cat, dog, *Alternaria alternata* , *Cladosporium herbarium* , grass pollen mix and tree pollen mix, cows' milk, soya, hens' egg, peanut and cod, plus positive and negative controls. A wheal diameter of 3 mm greater than the negative control indicated a positive allergen reaction. Individuals with at least one positive reaction were classified as atopic.

Spirometry at baseline was conducted on a group of 981 individuals at 10 years and 839 at age 18 using Koko spirometry software (Pds Instrumentation, Louisville, Ky) following ERS guidelines [13]. At 10 and 18 years, participants underwent methacholine BCT to evaluate AHR [14]. At 26 years, a small representative subgroup of participants who had undergone BCT at 10 and 18years were retested based on past/current wheeze status. The BCT utilised a Koko dosimeter (Pds Instrumentation) with a fixed straw, a compressed air source at 8 L/min and baffle position Devilbiss 646 nebuliser. Initially, a 0.9% saline inhalation was administered, followed by spirometry recording one minute later to establish a baseline measurement. Subsequently, incremental (doubling) concentrations of methacholine ranging from 0.0625 to 16 mg/mL were administered in serial fashion using 5 breaths at the dosimeter protocol. The Devilbiss 646 nebulisers were calibrated to deliver approximately 1.0 mL/min and were used in conjunction with the KoKo DigiDoser to deliver 5 breaths with an inhalation time of 5 s and breath holding for 5 s with firing times of 0.6 s, with 3 mL of solution in the nebuliser bowl, compressed air at 30 psi and a constant inspiratory flow rate of 0.5 L/s. Forced expiratory volume in 1 s (FEV_1_) was measured starting 1 min after completing dosing with each methacholine solution. A maximum of 5 FEV_1_ values were obtained over a 5‐min interval such that at least three values were within 5% of each other. The concentration at which a stable 20% decrease in FEV_1_ from the post saline value occurred was determined and expressed as PC_20_ FEV_1_ [14]. To participate in this test, participants needed to meet certain criteria, including being free from respiratory infections for at least 14 days, not having taken oral corticosteroids, refraining from using short‐acting beta_2_‐agonists for 6 h and long‐acting β2‐agonist medication for 48 h (Table S1). Methacholine BCT was undertaken in 57.0% (783/1373) at 10 years, 44.5% (585/1313) at age 18 years, and 8.3% (86/1034) at 26 years, using the same methodology (Figure S1).

Exhaled nitric oxide (FeNO) was measured using Niox mino, Aerocrine AB, Solna, Sweden, according to American Thoracic Society (ATS) guidelines [15]. A biofeedback mechanism was used to maintain the expiratory flow rate at 50 mL/s, and subjects exhaled against a resistance to prevent upper airway contamination. Up to two measurements were made in a standardised manner, with the subject standing without a nose clip. All measurements were undertaken before spirometric testing. The measurements were read from the plateau phase.

#### 2.1.1 Definitions

Asthma was defined as physician‐diagnosed asthma ever plus either current wheeze and/or on current asthma medications. Individuals with at least one positive reaction were classified as atopic. Current wheeze was defined as wheeze in the past 12 months. Rhinitis was defined by ‘have you ever had a problem with sneezing, runny or blocked nose in the absence of cold or flu’ plus ‘symptoms’ in the last 12 months. Indices used for spirometry included FEV_1_, forced expiratory volume (FVC), ratio of FEV_1_/FVC, and forced expiratory flow between 25 to 75% of the expiratory curve (FEF_25‐75_). We categorised AHR as ‘definite’ when PC_20_ FEV_1_ was < 4 mg/mL, following the categorisations outlined in the American Thoracic Society and European Respiratory Society AHR guidelines, which includes ‘Mild/Positive’ (1–4 mg/mL) and ‘Moderate–Severe’ (< 1 mg/mL) categories [14]. All those with > 4 mg/mL PC_20,_ including 4–16 mg/mL (borderline), and those not experiencing a 20% fall in FEV_1_ were regarded as negative. Persistent (AHR at both 10 and 18 years) and remittent (AHR at 10 but not at 18 years) AHR were calculated.

### 2.2 Data Analysis

Data were entered into SPSS (v24 IBM statistics, USA) using a double‐entry method and analysed using ‘The R Core Team (2023)’ [16].

We first assessed whether participants who underwent BCT at ages 10 and 18 differed from those in the whole cohort in asthma and allergy characteristics using one‐sample test for proportions. Then, we examined differences in the prevalence of AHR and other asthma characteristics at 10 and 18 years and associations of AHR with wheeze and asthma at these ages. We then investigated if changes in AHR across adolescence are also associated with changes in other asthma characteristics. Next, we studied the longitudinal evolution of AHR in the cohort by assessing proportions of those with persistent and remittent AHR from ages 10 to 18 years. We then analyzed early life risk factors reported in the literature for the development of asthma and allergic conditions to assess their relevance to the presence of AHR at ages 10 and 18 years. Finally, we extended longitudinal AHR examination to age 26 years in 86 participants who underwent AHR assessment at all three time points (10,18 and 26 years). All associations were tested using chi‐squared tests (or Fisher's exact test if the expected frequency in at least 20% of the cells were below 5) or McNemar test for paired comparisons for categorical variables, as appropriate. Continuous variables such as lung function were analysed using a *t*‐test for independent samples or a paired *t*‐test for paired samples, as appropriate. Statistical significance was set at *p* < 0.05.

## 3 Results

### 3.1 Prevalence of AHR

Participants who underwent BCT were broadly similar in demographic and atopic characteristics to the whole cohort (all *p* > 0.05) except those who had BCT at age 10, who had higher wheeze and asthma, while those who had BCT at 18 years had lower parental smoking at birth (Table S2). Of 783 participants who underwent BCT at 10 years, 169 (21.6%) had AHR (54.4% were male). Of 585 participants who underwent BCT at 18 years, 29 (5.0%) had AHR (48% were male).

### 3.2 AHR Association With Other Asthma Characteristics at Age 10 and 18 Years

**FIGURE 1:**
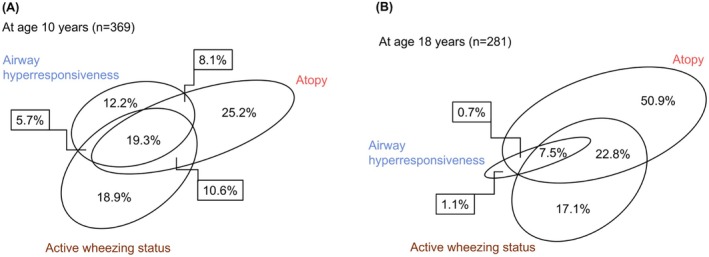
Venn diagram to demonstrate overlap in various characteristics of asthma; airway hyperresponsiveness, atopy and wheezing status with at least one positive condition for these three conditions. (A) Age 10 years (*n* = 369). (B) Age 18 years (*n* = 281).

At 10 years, 780 participants (of 783 who had BCT) had available information on AHR, wheezing status, and atopic status. Of these 780, 369 participants had at least one positive condition among AHR, wheezing status, or atopic status. Figure 1A depicts the overlaps (in %) among these characteristics at age 10; of the 369, 45 participants (12.2%) had AHR with no other asthma characteristics, 93 participants (25.2%) were identified as atopic‐only, while 70 participants (18.9%) were categorised as wheezing‐only.

At 18 years, 566 participants had data for AHR, wheezing and atopic status. Among them, 281 participants (49.7%) displayed at least one positive condition and were included in the analysis (Figure 1B); of these 281, 26 (9.3%) showed AHR, 230 (81.9%) were atopic and 133 (47.3%) individuals were current wheezers. Only 3 (1.1%) participants solely exhibited AHR, while 143 (50.9%) were solely atopic, and 48 (17.1%) participants were solely wheezing (Figure 1B).

### 3.3 Natural History of AHR From 10 to 18 Years

**FIGURE 2:**
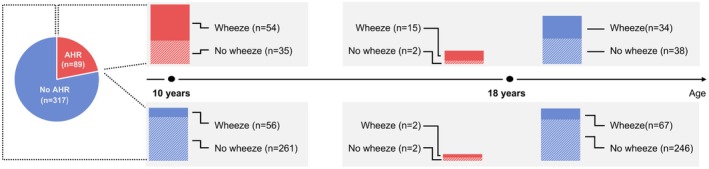
Longitudinal evolution of airway hyperresponsiveness among participants with bronchial challenge tests available at both 10 and 18 years (*n* = 406 at each age).

To assess the evolution of AHR from 10 to 18 years, we limited our analysis to the 406 participants who underwent BCT at both 10 and 18 years. Eighty‐nine of the 406 (21.6%) had AHR at age 10 Figure 2). Wheeze at age 10 was 60.7% (54/89) among those with AHR compared to 18.3% (56/317) without AHR (*p* < 0.001) (Table S3). At age 18, 81.0% (17/21) were wheezing among those with AHR compared to 26.2% (101/385) without AHR.

Of the 89 participants with AHR at 10 years, 72 (80.9%) became negative for AHR at 18 years, with only 17 participants (19.1%) retaining AHR between 10 years and 18 years (Figure 2). Conversely, among the 317 participants with negative AHR at 10 years, 313 (98.7%) remained negative for AHR at 18 years, with only 4 participants (1.3%) developing new AHR between 10 and 18 years; 2 of these reported wheezing at age 18.

### 3.4 Evolution of AHR in Comparison With Other Asthma Characteristics

During adolescence (between ages 10 and 18), AHR followed an opposite trajectory to other asthma characteristics, falling from 21.9% of participants at 10 years to 5.2% at 18 years, while current wheeze rose from 18.8% to 22.1%, asthma from 14.7% to 17.6%, atopy from 26.9% to 41.5% (all *p* < 0.01). Other factors associated with AHR, such as rhinitis, increased from 19.0% to 35.9%, and ~30% were smoking at age 18. Changes in lung function were less striking and contradictory, with an improvement in small airway function (FEF_25‐75_), while the FEV_1_/FVC ratio was reduced from 0.88 at 10 years to 0.87 at 18 years (*p* < 0.001; Table 1). At both 10 and 18 years, inhaled corticosteroid treatment was much higher in those with persistent AHR (69.2%), compared to remittent AHR (37.5%) AHR (Table 2). Further, there was no difference in inhaled corticosteroid treatment among those with no AHR at both 10 and 18 years: 12% (74/615) were treated with inhaled corticosteroids at age 10, compared to 11.1% at age 18 years (46/413, *p* = 0.70).

**TABLE 1:** Asthma‐associated characteristics of participants at age 10 and 18 years.

Participants with bronchial challenge tests	10 years	18 years	*p*
Airway hyperresponsiveness % (*n*/total *n*)	21.9% (89/406)	5.2% (21/406)	< 0.001
Atopy % (*n*/total *n*)	26.9% (202/751)	41.5% (312/751)	< 0.001
Wheeze % (*n*/total *n*)	18.8% (234/1243)	22.1% (275/1243)	0.009
Asthma	14.7% (181/1234)	17.6% (217/1234)	0.004
Inhaled corticosteroids	8.8% (80/905)	11.8% (107/905)	0.002
Rhinitis	19.0% (236/1241)	35.9% (445/1241)	< 0.001
Active smoking	0	28.8% (368/1278)	< 0.001
FEV_1_% predicted; mean (SD), *N* = 394	104.2 (11.2)	104.4 (12.0)	0.73
FVC % predicted; mean (SD), *N* = 393	103.1 (10.1)	103.0 (11.4)	0.70
FEF_25‐75_ (percent predicted); mean (SD)	102.8 (22.4)	105.8 (24.0)	< 0.001
FEV_1_/FVC ratio; mean (SD), *N* = 394	0.88 (0.06)	0.87 (0.07)	< 0.001

*Note:* McNemar paired tests were conducted to compare AHR, atopy, wheeze, asthma, and rhinitis. Lung function indices (FEV_1_, FVC, FEV_1_/FVC ratio and FEF_25‐75_) were compared using paired *t* tests.

**TABLE 2:** Comparison of participants with persistent versus remittent airway hyperresponsiveness (AHR) across adolescence for their asthma‐related characteristics.

Variable if categorical No (%) if numeric Mean (SD)	Participants with AHR at 10 and 18 years (persistent AHR) (*n* = 17)	Participants with AHR at 10 and negative at 18 years (remittent AHR) (*n* = 72)	*p* (persistent vs. remittent AHR)	Participants without AHR at 10 and 18 years (*n* = 313)
10 years				
Wheeze at 10 years	94.1% (16/17)	52.8% (38/72)	< 0.002	18.2% (57/313)
Asthma at 10 years	88.2% (15/17)	37.5% (27/72)	< 0.001	14.7% (46/313)
Inhaled corticosteroids (ICS) at 10 years	64.7% (11/17)	26.4% (19/72)	0.05	13.1% (41/313)
Atopic status at 10 years	88.2% (15/17)	55.6% (40/72)	0.01	22.1% (69/312)
FEV_1_/FVC	0.84 (0.08)	0.85 (0.06)	0.44	0.89 (0.06)
18 years				
Wheeze at 18 years	88.2% (15/17)	47.2% (34/72)	0.002	21.4% (67/313)
Asthma at 18 years	88.2% (15/17)	44.4% (32/72)	0.001	16.3% (51/313)
Inhaled corticosteroids (ICS) at 18 years	69.2% (9/13)	37.5% (18/48)	0.23	10.0% (22/221)
Atopic status at 18 years	100% (15/15)	60.0% (42/70)	0.002	37.4% (114/305)
FEV_1_/FVC	0.81 (0.07)	0.85 (0.07)	0.03	0.88 (0.06)

***Note:*** The subset of participants who were free from airway hyperresponsiveness (AHR) at the age of 10 and developed AHR at the age of 18 comprises only 4 and hence is not included. Two‐sample Student *t*‐tests were performed for each continuous variable. Chi‐square tests were performed for each categorical variable. *p* value for statistical significance was < 0.05.

There were statistically significant differences in asthma characteristics between persistent AHR and remittent AHR (Table 2). Participants with persistent AHR (*n* = 17) retained a high level of wheeze, asthma and atopy. However, the loss of AHR over adolescence did not result in the loss of all or most asthma characteristics. Among those 72 participants with remittent AHR, the proportion of wheeze (52.8% at age 10 vs. 47.2% at age 18; *p* = 0.7), asthma (37.5% at age 10% and 44.4% at age 18; *p* = 0.6) and use of inhaled corticosteroid (26.4% at age 10 vs. 37.5% at age 18, *p* = 0.4) remained the same (Table 2).

As the asthma/AHR relationship changes over adolescence, we limited our analysis to those without AHR and compared participants who wheezed to those who did not wheeze for their lung function status. Children who wheezed were similar to non‐wheezers in terms of spirometric characteristics of airway obstruction (FEV_1_, FEV_1_/FVC ratio and FEF_25‐75_) (all *p* > 0.05; Table 3) at age 10. However, at age 18, wheezy participants (without AHR) exhibited statistically significant evidence of airways obstruction and inflammation compared to non‐wheezers (*p* < 0.001; Table 3). However, when compared to participants with wheeze and AHR, they had less airway obstruction, atopy and information, but there was no difference in smoking and inhaled corticosteroid treatment (Table S4).

**TABLE 3:** Differences in airway obstruction and inflammation at age 10 and 18 years in non‐wheezer when compared to wheezer without airway hyperresponsiveness (AHR).

	Wheeze without AHR Mean (SD)	Non‐wheezer without AHR Mean (SD)	*p* *
At 10 years	*N* = 110	*N* = 775	
FVC (% predicted)	103.88 (9.45)	102.53 (10.63)	0.21
FEV_1_ (% predicted)	105.75 (10.97)	104.32 (11.22)	0.21
FEV_1_/FVC ratio	0.89 (0.06)	0.89 (0.05)	0.80
FEF_25‐75_ (% predicted)	104.77 (23.92)	104.40 (21.60)	0.87
At 18 years	*N* = 113	*N* = 427	
FVC (%predicted)	101.42 (11.47)	103.31 (1.96)	0.11
FEV_1_ (% predicted)	101.20 (11.98)	106.45 (11.17)	< 0.001
FEV_1_/FVC ratio	0.86 (0.08)	0.88 (0.06)	< 0.001
FEF_25‐75_ (% predicted)	100.29 (24.77)	110.49 (22.43)	< 0.001
BDR (%)^a^	6.19 (4.76)	4.37 (4.76)	< 0.001
FeNO (ppb)^b^	*N* = 70	*N* = 270	
	31.0 (45.0)	15.0 (13.0)	< 0.001

* *p* values were from two‐sample *t*‐tests.

^a^ BDR was calculated as % increase: Post‐bronchodilator – Pre‐bronchodilator/pre‐bronchodilator x 100.

^b^ Groups compared using Mann–Whitney *U*‐test. Medians and Interquartile ranges are displayed.

A sensitivity analysis using all data available at ages 10 and 18 years using different AHR cutoffs (4, 8 and 16 mg/mL) showed that the differences in prevalence and proportions of wheeze, atopy and FEV_1_% predicted between the two ages were similar and not statistically significant if we took different cutoffs (all *p* > 0.05; Table S5).

### 3.5 Early Life Risk Factors for AHR at Ages 10 and 18 Years

We found lower social class at birth as a protective factor and low birth weight and positive SPT at 1 year as the risk factors at univariate analysis only for AHR at age 10, which lost significance once adjusted for false discovery rate (Table S6). SPT positive at 2 years, recurrent wheezing, and SPT positive at 4 years remained statistically significant after adjustment for multiple testing. Similarly, for AHR at age 18 (Table S7), paternal asthma, formula feeding and recurrent wheeze and nasal symptoms at 4 years were significant at univariate testing only, while eczema and SPT positive at 4 years were the only risk factors that stood after multiple testing correction.

### 3.6 Natural History of AHR From 10 to 26 Years

**FIGURE 3:**
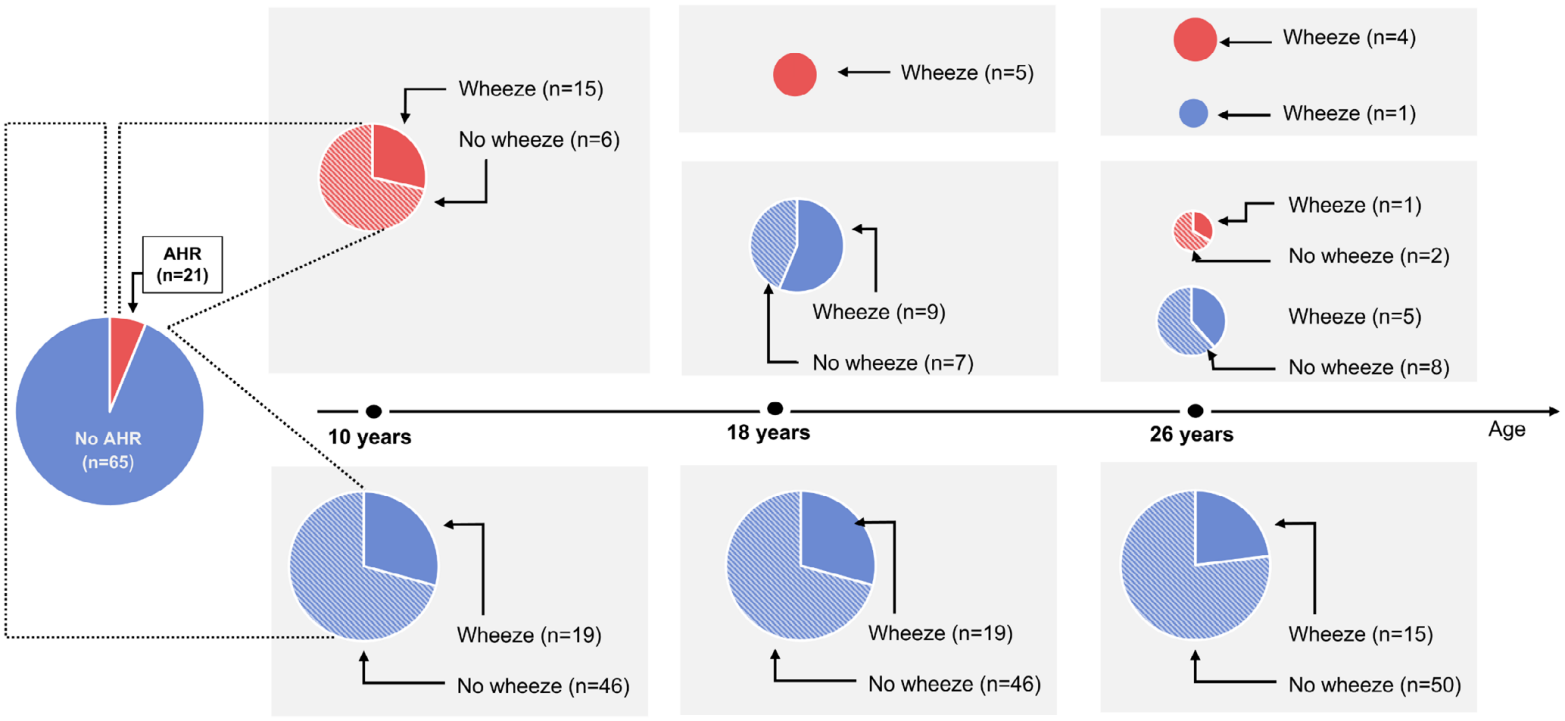
Sub‐group longitudinal evolution of airway hyperresponsiveness among participants with bronchial challenge tests available at 10, 18 and 26 years (*n* = 86).

A subgroup of 86 participants had bronchial challenges at three time points (ages 10, 18 and 26 years). Their descriptive characteristics are described in Table S8. Among these 86 participants, 65 (75.6%) were AHR negative at all three time points (Figure 3). Among the 21 participants with AHR at 10 years, only 5 (23.4%) were AHR positive at 18 years, all of whom were active wheezers at 18 years (100%). Four out of these five (80%) retained their AHR and wheeze status at age 26. Thus, among this small subset with information on AHR at all three time points, 4 participants were AHR positive at all three ages (4.7%), all of whom were wheezers at 26 years (100%).

## 4 Discussion

In this unselected population‐based cohort, 21.6% of children had AHR at age 10 years, while only 5% did so at age 18, with AHR improving in 81% of children between 10 and 18 years. Interestingly, this contrasts with asthma and its associated traits such as wheeze, atopy and rhinitis, which increased between 10 and 18 years. AHR became more closely associated with asthma over adolescence, with 61% of those with AHR wheezing at age 10, while 81% did so at age 18. However, loss of AHR was not associated with improvement in asthma‐related characteristics.

### 4.1 Prevalence of AHR

In our population, AHR during childhood was 21.6% with an equal sex distribution, which is consistent with other studies [17, 18]. However, at age 18, it was at the lower end (5%) of the range reported in population‐based studies (4% to 37%) although that depends on the cutoff used [11]. Some studies have found a change in the sex distribution of AHR where it becomes more common in females during adolescence, likely related to pubertal changes and hormonal effects [19]. With the use of modern dosimeters and nebulisers, PD_20_ is generally the preferred measure for AHR [7, 20]. However, there is a close correlation between PC_20_ and PD_20_ for any given testing protocol [4, 20] and cutoffs to define AHR have been better described for PC_20_. Hence, we preferred to use PC_20_ to define AHR. We used a 4 mg/mL cutoff in line with ATS and ERS recommendations to include mild to severe AHR (definite AHR) [4]. Although the use of a different cutoff such as 8 mg/mL or 16 mg/mL, changes absolute values and the percent who are positive at each age, it does not change the natural history of AHR or association with wheeze, atopy and lung function. Another variation to consider is the method used for BCT. We used methacholine, which is a direct test and most commonly used in asthma studies. Although it depends on the cutoff used, methacholine BCT is regarded as having a higher sensitivity, while using an indirect stimulus such as mannitol would yield a higher specificity.

### 4.2 The Evolution of AHR

Few studies have reported the evolution of AHR in an unselected population [21]. We observed a highly significant drop between 10 and 18 years; although the subgroup studied from 10 to 26 years indicates that AHR becomes more stable between 18 and 26 years with no further attrition. Inhaled corticosteroids suppress AHR, but there was no difference in the proportion treated with inhaled corticosteroids among those with no AHR at age 10 and 18, arguing against a strong treatment effect in AHR decline. Similarly, in addition to asthma, AHR is associated with rhinitis and smoking. However, we observed a higher prevalence of rhinitis and 30% taking up smoking during adolescence, and hence cannot explain AHR remission. Coates et al. recently showed that paediatric breathing pattern allows a greater pulmonary deposition of methacholine based on a μg/kg body weight, which might explain a higher prevalence of AHR, if the same criteria are used to define AHR in both adults and children [22]. Differences in airway calibre appearing during adolescence related to hormonal and developmental changes may provide further explanation of differences in BCT outcomes and their association with asthma [23].

Interestingly, AHR had a course independent of asthma with a diverging relationship between AHR and asthma‐associated traits as they evolved during adolescence, showing opposite trends except lung function, which showed no changes in FEV_1_ and FVC and improvement in small airways (FEF_25‐75_) (Table 1). We, and others, have previously shown that childhood AHR is associated with adverse lung function development independent of other asthma characteristics [18, 24, 25, 26, 27]. AHR may have a variable and a fixed component representing airway inflammation and remodelling, respectively [1]. It is possible that the association of AHR with lung function independent of asthma reflects the fixed component of AHR leading to airway remodelling. This hypothesis is supported by the seminal study of Grainge et al., who showed that bronchoconstriction, and not airway inflammation, induces airway remodelling [28].

Several studies have reported longitudinal consequences of childhood AHR with the development of asthma and lung function deficit even in asymptomatic children [18, 27, 29, 30]. Indeed, Palmer et al. showed that AHR in early infancy was significantly associated with lung function deficit and asthma diagnosis at 6 years of age [31]. We have previously shown that AHR at age 10 is associated not only with asthma and lung function deficit at age 10 but also at age 18, despite improving in the majority of participants [25]. Although the developmental period from childhood to adolescence is characterized by a dynamic and potentially reversible trajectory for AHR, there is no parallel improvement in asthma and lung function. Thus, children with childhood AHR should be targeted for preventive treatment. Among the early childhood risk factors for AHR, we only identified allergic symptoms and sensitisation in later childhood, which may serve as predictive markers to identify children at risk of AHR in later childhood for preventive strategies.

### 4.3 AHR and Asthma

Although AHR is regarded as a cardinal feature of asthma, the relationship of AHR with asthma is complex [1, 11]. Asthma is a syndrome comprising traits such as wheezing, airway inflammation, airway obstruction and AHR. As we have shown (Figure 1), the overlap between these characteristics is partial at best. Further, participants with AHR did not show significantly higher BDR compared to those without AHR (Table S2). This is supported by a recent study of asthma patients, where no correlation was found between methacholine AHR and BDR and AHR outperformed BDR in asthma diagnosis [6]. Thus, AHR assumes significance as a definitive test in symptomatic individuals where doubt remains regarding diagnosis [7]. It is argued that, as AHR is not specific for asthma, its value is in ‘ruling out asthma’ when negative [1]. In our study, the relationship of AHR with asthma symptoms was highly age dependent, and we argue that age should be taken into account when interpreting AHR as a defining trait for asthma diagnosis. At age 10 years, AHR could be used to rule out asthma diagnosis as 110 wheezy children with no AHR were similar to non‐wheezers in terms of lung function (Table 3). However, at age 18, AHR should be used to ‘rule in’ asthma as participants with AHR had definite asthma (88% had diagnosed asthma and 70% were taking inhaled corticosteroids). On the other hand, nearly half of the participants who lost AHR at 18 years were still wheezing, over half were atopic, they had impaired lung function, and many were taking asthma treatment (Table 2). Further, participants with wheeze and no AHR were significantly different in asthma characteristics from non‐wheezers and thus the absence of AHR in these wheezy young adults cannot be used to ‘rule out’ asthma (Table 3). However, wheeze is heterogeneous, and participants who wheeze without AHR may have other pathologies. When compared to the wheeze with AHR, wheezers without AHR did have less asthma and atopy, but smoking prevalence and inhaled steroid treatment were high in both groups and not statistically different. We have previously shown that 5% of participants at age 18 wheeze without a diagnosis of asthma, and yet had high treatment need with smoking as a major risk factor [32]. We used an AHR threshold of PC20 < 4 mg/mL as the value defining the presence of ‘definite’ AHR at both ages 10 and 18 years [14]. However, as the relationship of AHR with asthma changes over time, we may need to use varying, age‐based cutoffs to define positive value [21].

*Strengths and Limitations:* A key strength of our study is the longitudinal dataset with high retention and a large number of participants assessed in detail for asthma characteristics, including AHR. A limitation is that the study population exhibits a high degree of homogeneity, underscoring the need for replication in diverse populations. Wheeze and asthma were higher among those who attended for AHR assessment (Table S2), possibly inflating AHR prevalences but unlikely to have changed the natural history trends or other associations. FeNO was not available at age 10 as this was not a routine test at that time, and we did not have eosinophils as indicators of type 2 airway inflammation. AHR data were limited at age 26 years due to a lack of adequate funding.

## 5 Conclusion

The natural evolution of AHR is independent, and often in the opposite direction to other characteristic traits of asthma, such as wheezing and atopy. Thus, age should be taken into consideration when interpreting AHR. By unravelling the complexities of AHR evolution, we can refine our understanding of asthma's trajectory and enhance strategies for personalised management.

## Author Contributions

S.H.A. conceptualised the study. S.C. performed data analysis, guided by S.H.A., R.J.K. and H.Z. S.C. and S.H.A. wrote the first draft. The text was then submitted to R.J.K. and H.Z. for review and editing, and revised accordingly. All authors have read and agreed to the final submitted draft.

## Conflicts of Interest

The authors declare no conflicts of interest.

## Supporting information


**Appendix S1.** Supporting Information.

**Appendix S2.** Supporting Information.

## Data Availability

The data that support the findings of this study are available on request from the corresponding author. The data are not publicly available due to privacy or ethical restrictions.
