# Supplementary material for: Natural History of Airway Hyperresponsiveness and Its Association With Asthma Traits
Source: Allergy. 2025 Aug 22;81(1):121–9. doi: 10.1111/all.70006 (PMC12773667; doi:10.1111/all.70006)
Supplement: Supplementary file 1 — Appendix S1. Supporting Information. [file ALL-81-121-s002.pdf]

CONSORT
TRANSPARENT REPORTING of TRIALS

CONSORT 2010 Flow Diagram

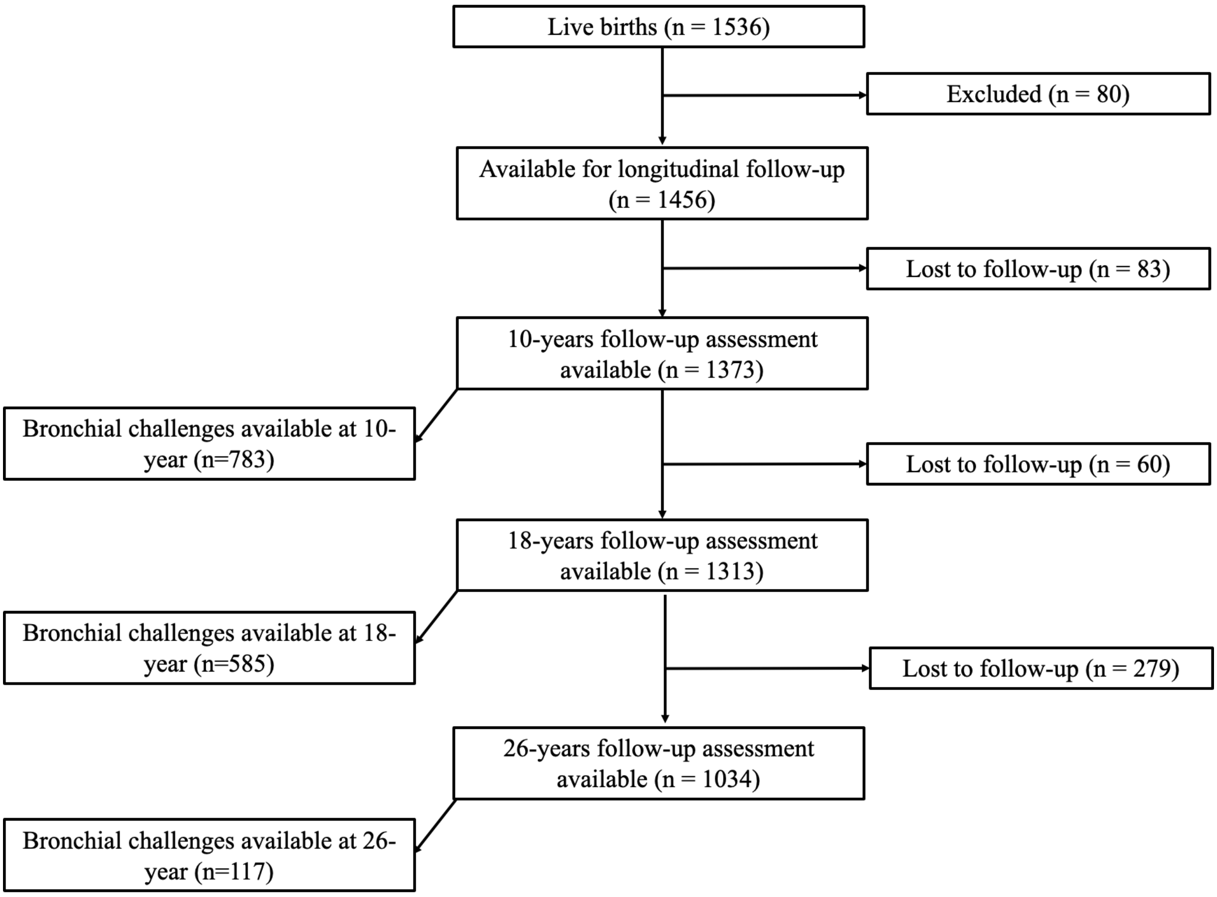

Figure E1. Flow chart of the study design
