# Supplementary material for: Natural History of Airway Hyperresponsiveness and Its Association With Asthma Traits
Source: Allergy. 2025 Aug 22;81(1):121–9. doi: 10.1111/all.70006 (PMC12773667; doi:10.1111/all.70006)
Supplement: Supplementary file 2 — Appendix S2. Supporting Information. [file ALL-81-121-s001.docx]

#### **On-line supplement**

**Title: Natural history of airway hyperresponsiveness and its association with asthma traits**

#### **Methods**

**Risk Factors collection and definitions**

Following information was collected from questionnaires administered soon after birth of the child and/or collected from maternity records (as appropriate) and updated where relevant at subsequent assessments at ages 1, 2 and 4 years.

**Sex:** Biological sex determined at birth from maternity files.

**Maternal asthma:** A yes response to *“have you suffered from asthma or wheezing attacks, which was diagnosed by a doctor and/or treated for it?”*

**Paternal asthma:** *“*A yes response to *“has your partner suffered from asthma or wheezing attacks, which was diagnosed by a doctor and/or treated for it?”*

**Maternal smoking during pregnancy/at birth:** A yes response to *“do you smoke in the house?”*

**Any smoking during pregnancy/at birth:** A yes response to “*does anyone smoke in the house?*”

**Pets (cat/dog) exposure during pregnancy/at birth**: A yes response to *“any pets in the house”?* and if yes, to state that they have either cat or dog or both.

**Social class (lower) during pregnancy:** Social class was determined using British Registrar General classification which is based on parental occupation of 1-6 social classes (1-3 was regarded as upper social class, 4-6 as lower social class).

**Caesarean section:** Yes or no, collected from maternity records.

**Low birth weight:** Low (<2.5kg), normal (≥2.5kg), collected from maternity records.

**High cord IgE (≥0.5):** Cord blood was routinely collected at the time of birth and this was analysed for cord serum total IgE.

**Method of feeding:** Questionnaire were completed regarding method of feeding at ages 1 and 2, seeking information on duration of breast feeding, age of introduction of formula feeding and age of introduction of solids food. The duration of total breast feeding (≥3 months), exclusive breast feeding (≥3 months), and early formula feeding (<6 months)

were drawn from this information.

**Smoking exposure during year 1 and 2:** A yes response to “*does anyone smoke in the house?”*

**Recurrent chest (≥2) infections at 1 or 2 years:** A yes response to *“Has your child had recurrent (>2 in the last 12 months) chest infections in the last 12 months”.* Information obtained at both 1 and 2 years and data were combined to analyse this variable.

**Overweight at 4 years:** BMI calculated at 4 years from height and weight information.

**Method of cooking (Gas) at 4 years:** Questionnaire information; “*what is your method of cooking (i) gas, (ii) electric, (iii) both?”*

**Early life clinical manifestation:**

**Recurrent Wheeze year 1:** *”Has your child had wheezing episodes”* (i) yes or no. If yes, *“was this <3, ≥3 or frequent*”. Recurrent wheeze was defined as children having ≥3 or frequent wheezing episodes in the last 12 months.

**Eczema year 1, 2 and 4 years:** questionnaire information with a yes to both *“(i) ever eczema, plus (ii) presence of an itchy rash during the previous 12 months”*

**Recurrent Nasal symptoms at 1, 2 and 4 years:** yes to the question, *“has you child had recurrent nasal congestion?”*

**Skin test positive (≥ 1 allergen) at year 1, 2 and 4 years:** as defined in the main manuscript for later time points.

#### **Supplementary tables**

**Table 1: Medications and substances to stop before methacholine testing**

| **Factor** | **Time before test** |
| --- | --- |
| **Foods**  Chocolate  Coffee and tea  All caffeine containing beverages such as coke | Day of study |
| **Medications**   1. Antihistamines 2. Leukotriene modifiers such as montelukast 3. Cromolyn sodium 4. Nedocromil 5. Short-acting inhaled anticholinergic agents such as ipratropium 6. Long-acting inhaled anticholinergic agents such as tiotropium 7. Short-acting β-agonist bronchodilators such as salbutamol, fenoterol 8. Long-acting β-agonist bronchodilators such as salmeterol, formoterol 9. Combination of inhaled long-acting β-agonist and corticosteroids such as salmeterol/fluticasone 10. Theophyllines | 72 hours  24 hours  8 hours  48 hours  24 hours  96 hours  8 hours  48 hours  48 hours  48 hours |

**Table E2.** Comparison of participants who had bronchial challenge test (BCT) at 10 and 18-years with the whole cohort.

| **Variable**  **Number (%)** | **Participants with BCT at 10 (n=783)** | **P-value** | **Total cohort**  **(n=1536)** | **Participants with BCT at 18**  **(n=585)** | **P-value** |
| --- | --- | --- | --- | --- | --- |
| **Male sex** | 51.5%  (403/783) | 0.90 | 51.2% (786/1536) | 49.9%  (292/585) | 0.60 |
| **Low birth weight** | 3.9%  (30/777) | 0.54 | 4.4% (66/1497) | 3.9%  (22/570) | 0.31 |
| **Low Socio-economic level** | 14.2%  (111/777) | 0.55 | 15.4% (209/1357) | 13.4%  (77/574) | 0.33 |
| **Maternal asthma** | 12.2%  (95/779) | 0.40 | 10.9% (165/1517) | 10.8%  (63/584) | 0.95 |
| **Paternal asthma** | 10.2%  (79/773) | 0.83 | 9.9%  (149/1504) | 9.9%  (57/578) | 0.98 |
| **Maternal smoking at birth** | 22.4%  (173/779) | 0.11 | 26.0% (393/1509) | 18.1%  (105/579) | 0.003 |
| **Paternal smoke at birth** | 38.0%  (294/774) | 0.45 | 40.5% (608/1503) | 32.9%  (191/580) | 0.03 |
| **Pets at home at birth** | 58.0%  (451/778) | 0.40 | 56.1% (850/1514) | 56.7%  (330/582) | 0.82 |
| **Recurrent chest infections (year 1)** | 8.6%  (63/736) | 0.36 | 7.4% (101/1359) | 7.1%  (39/551) | 0.79 |
| **Positive SPT (year 4)** | 23.3%  (144/617) | 0.15 | 19.7%  (192/980) | 19.8%  (96/485) | 0.96 |
| **Wheeze at 10-years** | 26.0%  (203/783) | 0.002 | 18.9% (259/1373) | 22.1%  (127/575) | 0.19 |
| **Asthma at 10-years** | 19.9%  (156/781) | 0.008 | 14.7% (201/1368) | 17.4%  (100/575) | 0.20 |
| **Atopic status at 10-years** | 29.9%  (233/780) | 0 .16 | 26.9% (279/1036) | 27.0%  (146/540) | 0.94 |
| **Wheeze at 18-years** | 28.6%  (208/727) | 0.01 | 22.4% (294/1312) | 23.6%  (138/584) | 0.56 |
| **Asthma at 18-years** | 23.4%  (169/723) | 0.01 | 17.7% (231/1305) | 19.3%  (113/585) | 0.40 |
| **Atopic status at 18-years** | 44.0%  (244/555) | 0.31 | 41.2%  (351/851) | 40.7%  (231/567) | 0.85 |

Note: One sample test for proportion were performed for each categorical characteristic to assess if those who underwent methacholine BCT are different in key characteristics to the whole cohort.

*P-value for significance is 0.05.

**Table E3.** Characteristics of the 406 included participants at 10 and 18-years stratified by 10-year airway hyperresponsiveness (AHR) status.

| **Variable**  **If categorical**  **% (n/N)**  **If numeric**  **Mean (SD)** | **Participants with AHR at 10-years** | **Participants without AHR at 10-years** | **P-value** |
| --- | --- | --- | --- |
| **Sex**  **Male** | 50.6%  (45/89) | 50.0%  (158/316) | 0.93 |
| **Low socioeconomic level at 10-years** | 13.6%  (12/88) | 22.2%  70/316 | 0.19 |
| **Passive tobacco smoking at 10-year** | 31.2%  (24/77) | 41.4%  118/285 | 0.10 |
| **Wheeze at 10-years** | 60.7%  (54/89) | 18.3%  56/317 | <0.001 |
| **Asthma at 10-years** | 47.2%  (42/89) | 14.8%  (47/317) | <0.001 |
| **Rhinitis at 10-years** | 34.8%  (31/89) | 17.8%  (56/315) | <0.001 |
| **Eczema at 10-years** | 28.1%  (25/89) | 19.6%  62/316 | 0.09 |
| **Atopic status at 10-years** | 61.8%  (55/89) | 22.2%  (70/316) | <0.001 |
| **Inhaled corticosteroids at 10-years** | 50.0%  (43/86) | 14.8%  (47/317) | <0.001 |
| **FEV_1_ (L)**  **Mean**  **(sd)** | 1.91  (0.25) | 2.04  (0.32) | <0.001 |
| **FVC (L)**  **Mean**  **(sd)** | 2.24  (0.28) | 2.30  (0.37) | 0.12 |
| **FEV_1_/FVC (%)**  **Mean**  **(sd)** | 0.85  (0.07) | 0.89  (0.06) | <0.001 |
| **FEF_25-75_**  **Mean**  **(sd)** | 2.09  (0.54) | 2.47  (0.54) | <0.001 |
|  | **Participants with AHR at 18-years** | **Participants without AHR at 18-years** | **P-value** |
| **Sex (Male)** | 47.6% (10/21) | 50.1% (193/385) | 1.0 |
| **Smoking (personal)** | 29.4% (5/17) | 28.3% (81/286) | 1.0 |
| **Wheeze at 18-years** | 81.0%  (17/21) | 26.2%  (101/385) | <0.001 |
| **Asthma at 18-years** | 81.0%  (17/21) | 21.6%  (83/385) | <0.001 |
| **rhinitis at 18-years** | 90.5%  (19/21) | 41.3%  (159/385) | <0.001 |
| **Eczema at 18-years** | 28.6%  (6/21) | 10.9%  (42/385) | 0.03 |
| **Atopic status at 18-years** | 89.5%  (17/19) | 41.6%  (156/375) | <0.001 |
| **Inhaled corticosteroids at 18-years** | 68.8%  (11/16) | 14.9%  (40/269) | <0.001 |
| **FEV_1_ (L)**  **Mean (SD)** | 3.52 (0.92)  N=19 | 4.05 (0.80)  N=376 | 0.006 |
| **FVC (L)**  **Mean (SD)** | 4.43 (1.03)  N=19 | 4.64 (0.92)  N=376 | 0.35 |
| **FEV_1_/FVC (%)**  **Mean (SD)** | 0.79 (0.08)  N=19 | 0.88 (0.07)  N=376 | <0.001 |
| **FEF_25-75_**  **Mean (SD)** | 3.23 (1.03)  N=19 | 4.50 (1.20)  N=376 | <0.001 |
| **BDR** | 8.49 (6.61)  N=19 | 4.91 (4.96)  N=376 | 0.06 |
| **FeNO**  **Mean (SD)** | 58.00 (39.0)  N=19 | 28.24 (24.07)  N=364 | <0.001 |

Note: Chi-square tests were performed for each categorical variables.

Two-sample student t-tests were performed for each continuous variables.

**Table E4:** Comparison of participants at age 18 who wheezed with AHR and those who were wheezing but did not have AHR for their asthma related characteristics.

| **Variable If categorical**  **No (%)**  **If numeric Mean (SD)** | **Wheeze with AHR** | **Wheeze without AHR** | **P-value** |
| --- | --- | --- | --- |
| **Asthma at 18-years** | 95.2% (20/21) | 75.2% (88/117) | 0.04 |
| **Rhinitis at age 18** | 95.2% (20/21) | 63.2 (74/117) | 0.004 |
| **Atopy at age 18** | 100% (21/21) | 57.1% (64/112) | <0.001 |
| **Inhaled corticosteroids** | 66.7% (12/18) | 61.5% (40/65) | 0.69 |
| **Smoking at age 18** | 28.6 (6/21) | 26.5 (32/113) | 0.84 |
|  | N=21 | N=113 |  |
| **FEV_1_ % predicted** | 92.0 (14.8) | 101.2 (11.9) | 0.002 |
| **FVC % predicted** | 98.3 (11.6) | 101.4 (11.5) | 0.25 |
| **FEV_1_/FVC ratio** | 0.80 (0.08) | 0.86(0.08) | <0.001 |
| **FEF_25-75_** | 78.7 (22.4) | 100.3 (24.8) | <0.001 |
| **FeNO (ppb)#** | 84.5 (59.0) | 31.0 (45.0) | <0.001 |

Two sample student t-tests were performed for each continuous variable.

Chi-square tests were performed for each categorical variable.

P-value for statistical significance was < 0.05.

#Groups compared using Mann-Whitney U test. Medians and Interquartile ranges are displayed

|  | 4mg/ml |  | 8mg/ml | 16mg/ml |  |
| --- | --- | --- | --- | --- | --- |
| **Prevalence of AHR** | | | | | |
| **10 years** | 21.6% (169/783) | P= 1.0 | 30.1% (236/783) | P= 0.7 | 41.8% (327/783) |
| **18 years** | 5.0% (29/585) |  | 7.5% (44/585) |  | 11.8% (69/585) |
| **Wheezing among those with AHR** | | | | | |
| **10 years** | 55.0% (93/169) | P= 0.6 | 47.9% (113//169) | P= 1.0 | 41.9% (137/327) |
| **18 years** | 72.4% (21/29) |  | 72.7 (32/44) |  | 63.8% (44/69) |
| **Atopy among those with AHR** | | | | | |
| **10 years** | 60.0% (100/167) | P= 0.7 | 53.8% (126/234) | P= 0.7 | 46.2% (150/325) |
| **18 years** | 88.5% (23/26) |  | 87.5% 35/40) |  | 76.6% (49/64) |
| **FEV_1_ % predicted (Mean, SD) among those with AHR** | | | | | |
| **10 years** | 94.7 (11.3) | P= 1.0 | 95.5 (11.3) | P= 1.0 | 96.5 (11.3) |
| **18 years** | 93.9 (14.2) |  | 95.9 (13.9) |  | 98.4 (12.7) |

**Table E5:** Sensitivity analysis to show that the pattern of changes in prevalence, and proportions of wheezing, atopy and lung function among those with AHR based on different cutoffs is similar between ages 10 and 18 when taking different cutoffs for AHR.

Note: One sample proportion test were carried to compare the difference between 10 and 18 years when taking 4mg/ml versus 8mg/ml, and 8mg/ml versus 16mg/ml cutoff in PC_20_ methacholine.

**Table E6.** Early life risk factors for AHR at 10 years (univariate analysis).

| **Variable**  **If categorical; % (n/N)**  **If numeric; Mean (SD)** | **Participants with AHR at 10-years** | **Participants without AHR at 10-years** | **Odds ratio (95% CI)** | **P-value** |
| --- | --- | --- | --- | --- |
| **Early life factors** | | | | |
| **Male** | 55  (93/169) | 50.6  (311/615) | 0.8  (0.6-1.2) | 0.3 |
| **Maternal asthma** | 13.0  (22/169) | 11.9  (73/611) | 1.1  (0.7-1.8) | 0.7 |
| **Paternal asthma** | 14.3  (24/168) | 9.2  (56/607) | 1.6  (1.0-2.7) | 0.06 |
| **Maternal smoking during pregnancy** | 21.6  (36/167) | 22.8  (138/606) | 1.1  (0.7-1.6) | 0.7 |
| **Any smoking during pregnancy** | 44.3  (74/167) | 42.5  (254/597) | 0.9  (0.7-1.3) | 0.7 |
| **Cat exposure during pregnancy** | 30.2  (51/169) | 33.4  (204/611) | 1.1  (0.8-1.7) | 0.5 |
| **Dog exposure during pregnancy** | 30.2  (51/169) | 30.3  (185/611) | 1.0  (0.7-1.5) | 1.0 |
| **Social class (lower) during pregnancy** | 53.9  (55/102) | 40.9  (156/381) | 0.6  (0.4-0.9) | 0.02 |
| **Caesarean section** | 12.7  (7/55) | 6.7  (13/193) | 2.0  (0.8-5.3) | 0.1 |
| **Low birth weight** | 7.3  (12/165) | 3.0  (18/598) | 2.5  (1.2-5.4) | 0.01 |
| **High cord IgE (≥0.5)** | 16.5  (21/127) | 11.3  (53/468) | 1.5  (0.9-2.7) | 0.1 |
| **Early formula feed**  **(<6 months)** | 60.8  (90/148) | 63.0  (344/546) | 0.9  (0.6-1.3) | 0.6 |
| **Breast fed**  **(≥3 months)** | 53.2  (82/154) | 53.7  (308/574) | 1.0  (0.7-1.4) | 0.9 |
| **Exclusive breast feeding**  **(≥3 month)** | 34.5  (51/148) | 32.8  (176/537) | 1.1  (0.7-1.6) | 0.7 |
| **Smoking exposure during year 1** | 44.3  (74/167) | 42.5  (254/597) | 0.9  (0.7-1.3) | 0.7 |
| **Smoking exposure during year 2** | 40.1  (59/147) | 41.2  (218/529) | 1.0  (0.7-1.4) | 0.4 |
| **Recurrent chest (≥2) infections at 1 or 2 years** | 20.8  (31/149) | 20.1  (108/538) | 1.0  (0.7-1.6) | 0.8 |
| **Overweight at 4 years** | 19.5  (33/169) | 23.1  (142/615) | 0.8  (0.5-1.2) | 0.3 |
| **Method of cooking (Gas) at 4 years** | 75.6  (124/164) | 72.0  (437/607) | 1.2  (0.8-1.8) | 0.4 |
| **Early life clinical manifestation** | | | | |
| **Recurrent Wheeze year 1** | 12.7  (20/158) | 11.8  (69/587) | 1.1  (0.6-1.9) | 0.8 |
| **Eczema year 1** | 13.3  (21/158) | 11.1  (65/587) | 1.2  (0.7-2.1) | 0.4 |
| **Recurrent Nasal symptoms** | 8.9  (14/158) | 11.2  (66/587) | 0.8  (0.4-1.4) | 0.4 |
| **Skin test positive**  **(≥ 1 allergen) at year 1** | 37.5  (15/40) | 22.8  (39/171) | 2.0  (1.0-4.2) | 0.05 |
| **Recurrent Wheeze year 2** | 15.4  (23/149) | 13.8  (75/542) | 1.1  (0.7-1.9) | 0.6 |
| **Eczema year 2** | 13.4  (20/149) | 11.3  (61/542) | 1.2  (0.7-2.1) | 0.5 |
| **Recurrent Nasal symptoms at year 2** | 10.1  (15/149) | 12.7  (69/542) | 0.8  (0.4-1.4) | 0.4 |
| **Skin test positive**  **(≥ 1 allergen) at year 2** | **34.4**  **(21/61)** | **14.1**  **(31/220)** | **3.2**  **(1.7-6.1)** | **<0.001** |
| **Recurrent Wheeze year 4** | **32.0**  **(47/147)** | **16.0**  **(88/551)** | **2.5**  **(1.6-3.7)** | **<0.001** |
| **Eczema year 4** | 19.0  (28/147) | 10.2  (56/551) | 2.1  (1.3-3.4) | 0.003 |
| **Recurrent Nasal symptoms at year 4** | 9.5  (14/148) | 6.0  (33/552) | 1.6  (0.9-3.2) | 0.1 |
| **Skin test positive**  **(≥ 1 allergen) at year 4** | **50.7**  **(68/134)** | **15.9**  **(77/484)** | **5.4**  **(3.6-8.3)** | **<0.001** |

Notes: Risk factors are the factors during childhood, which may have individual risk of developing AHR at age 10. To identify the statistically significant factors among various potential factors, Chi-square tests were applied (Fisher’s exact test if the frequency in at least 20% of the cells were below 5). Risk factor showing statistical significance are in bold font, after adjusting for multiple testing using the Bonferroni approach (adjusted significance level = 0.05/31=0.0016).

**Table E7.** Early life risk factors for AHR at 18-years (univariate analysis).

| **Variable**  **If categorical; % (n/N)**  **If numeric; Mean (SD)** | **Participants with AHR at 10-years** | **Participants without AHR at 10-years** | **Odds ratio (95% CI)** | **P-value** |
| --- | --- | --- | --- | --- |
| **Male** | 48.3  (14/29) | 50.0  (278/556) | 1.1  (0.5-2.2) | 0.9 |
| **Maternal asthma** | 13.8  (4/29) | 10.7  (59/553) | 1.3  (0.5-4.0) | 0.6 |
| **Paternal asthma** | 20.7  (6/29) | 9.3  (51/549) | 2.5  (1.0-6.5) | 0.05 |
| **Maternal smoking during pregnancy** | 20.7  (6/29) | 18.0  (99/550) | 0.8  (0.3-2.21) | 0.6 |
| **Any smoking during pregnancy** | 44.8  (13/29) | 35.7  (194/543) | 0.7  (0.3-1.5) | 0.3 |
| **Cat exposure during pregnancy** | 24.1  (7/29) | 34.9  (193/553) | 1.7  (0.7-4.0) | 0.2 |
| **Dog exposure during pregnancy** | 27.6  (8/29) | 26.8  (148/553) | 1.0  (0.4-2.2) | 1.0 |
| **Social class (upper) during pregnancy** | 46.7  (7/15) | 52.0  (171/329) | 0.8  (0.3-2.3) | 0.7 |
| **Caesarean section** | 11.1  (1/9) | 9.7  (15/154) | 1.2  (0.1-9.9) | 0.9 |
| **Low birth weight** | 3.4  (1/29) | 3.9  (21/541) | 0.9  (0.1-6.8) | 0.9 |
| **High cord IgE (≥0.5)** | 9.1  (2/22) | 11.5  (52/454) | 0.8  (0.2-3.4) | 0.7 |
| **Early formula feed**  **(<6 months)** | 77.8  (21/27) | 58.0  (287/495) | 2.5  (1.0-6.4) | 0.04 |
| **Breast fed**  **(≥3 months)** | 35.7  (10/28) | 21.6  (267/517) | 1.9  (0.8-4.2) | 0.1 |
| **Exclusive breast feeding**  **(≥3 month)** | 21.4  (6/28) | 37.6  (183/487) | 0.5  (0.2-1.1) | 0.08 |
| **Smoking exposure during year 1** | 35.7  (10/28) | 31.9  (167/524) | 1.2  (0.5-2.6) | 0.7 |
| **Smoking exposure during year 2** | 33.3  (8/24) | 33.6  (163/485) | 1.0  (0.4-2.4) | 1.0 |
| **Recurrent chest (≥2) infections at 1 or 2 years** | 20.8  (5/24) | 17.3  (85/491) | 1.3  (0.5-3.5) | 0.7 |
| **Overweight at 4 years** | 19.2  (5/26) | 21.6  (111/515) | 0.9  (0.3-2.4) | 0.8 |
| **Method of cooking (Gas) at 4 years** | 71.4  (20/28) | 70.9  (380/536) | 1.0  (0.4-2.4) | 1.0 |
|  | **Early life clinical manifestation** | | | |
| **Recurrent Wheeze year 1** | 10.7  (3/28) | 9.1  (48/528) | 1.2  (0.3-4.1) | 0.7 |
| **Eczema year 1** | 21.4  (6/28) | 10.8  (57/528) | 2.3  (0.9-5.8) | 0.08 |
| **Recurrent Nasal symptoms** | 10.7  (3/28) | 10.2  (54/528) | 1.1  (0.3-3.6) | 0.9 |
| **Skin test positive**  **(≥ 1 allergen) at year 1** | 22.2  (2/9) | 24.5  (35/143) | 0.9  (0.2-4.4) | 0.9 |
| **Recurrent Wheeze year 2** | 12.0  (3/25) | 11.4  (57/498) | 1.1  (0.3-3.6) | 0.9 |
| **Eczema year 2** | 20.0  (5/20) | 10.2  (51/498) | 2.2  (0.8-6.1) | 0.1 |
| **Recurrent Nasal symptoms at year 2** | 12.0  (3/25) | 10.6  (53/498) | 1.1  (0.3-4.0) | 0.8 |
| **Skin test positive**  **(≥ 1 allergen) at year 2** | 23.1  (3/13) | 14.4  (28/194) | 1.8  (0.5-6.9) | 0.4 |
| **Recurrent Wheeze year 4** | 34.6  (9/26) | 15.4  (79/514) | 2.9  (1.3-6.8) | 0.01 |
| **Eczema year 4** | **34.6**  **(9/26)** | **11.1**  **(57/513)** | **4.2**  **(1.8-9.9)** | **<0.001** |
| **Recurrent Nasal symptoms at year 4** | 15.4  (4/26) | 5.3  (27/513) | 3.3  (1.1-10.2) | 0.03 |
| **Skin test positive**  **(≥ 1 allergen) at year 4** | **66.7**  **(16/24)** | **17.4**  **(80/461)** | **9.5**  **(3.9-23.0)** | **<0.001** |

Notes: Risk factors are the factors during childhood, which may have individual risk of developing AHR at age 18. To identify the statistically significant factors among various potential factors, Chi-square tests were applied (Fisher’s exact test if the frequency in at least 20% values in a cell were below 5). Risk factor showing statistical significance are in bold font, after adjusting for multiple testing using the Bonferroni approach (adjusted significance level = 0.05/31=0.0016).

**Table E8.** Characteristics of 86 participants with bronchial challenge test available at 3 ages (10, 18 and 26-year)

|  | **10-years** | **18-years** | **26-years** |
| --- | --- | --- | --- |
| **Male sex**  **N° (%)** | 46.5% (40/86) | | |
| **Atopic status** | 36.1% (31/86) | 46.5% (40/86) | 55.8% (48/86) |
| **Wheeze** | 39.5% (34/86) | 38.4% (33/86) | 30.2% (26/86) |
| **Asthma** | 37.2% (32/86) | 32.6% (28/86) | 20.9% (18/86) |
| **Inhaled Corticosteroid use** | 65.2% (30/46) | 38.1% (16/42) | 21.1% (4/19) |
